# Tuberculous Osteomyelitis in the Hand

**DOI:** 10.7759/cureus.99198

**Published:** 2025-12-14

**Authors:** Andres Sanchez Nadales, Mustafa Alnuaimi, Antonio Crespo, Steve Carlan

**Affiliations:** 1 Internal Medicine, Orlando Regional Medical Center, Orlando, USA; 2 Infectious Diseases, Orlando Regional Medical Center, Orlando, USA; 3 Research and Academic Affairs, Orlando Regional Medical Center, Orlando, USA

**Keywords:** extrapulmonary tb, hand trauma, metacarpal infection, mycobacterium tuberculous, tb osteomyelitis

## Abstract

Tuberculous osteomyelitis is recognized as a rare form of extrapulmonary tuberculosis, particularly when involving the small bones of the hand in adults. Early diagnosis is challenging due to its uncommon presentation and nonspecific symptoms. These factors often contribute to the delayed identification and management of the condition.

We report a 22-year-old male construction worker from Mexico who presented with swelling and pain localized to his right hand. One week prior to presentation, he experienced minor blunt trauma to the fifth metacarpal region when a piece of wood struck his hand, with no skin breach. He denied any systemic symptoms such as fever or weight loss. Additionally, there was no history of incarceration, overcrowded living conditions, or known tuberculosis exposure. Imaging studies revealed extensive pathology in the chest and hand. Bone and soft tissue samples from the hand tested positive for acid-fast bacteria (AFB) and *Mycobacterium tuberculosis* (TB) by polymerase chain reaction (PCR). This case highlights an unusual presentation of tuberculous osteomyelitis involving a small hand bone in a young, immunocompetent patient.

The report emphasizes the importance of comprehensive diagnostics when evaluating patients with localized swelling and no clear entry site. Tuberculosis should be considered in the differential diagnosis even without systemic symptoms.

## Introduction

Tuberculosis (TB) remains a major global health issue, with extrapulmonary manifestations (EPTB) making up a significant portion of cases [1]. Skeletal tuberculosis, a rare form of EPTB, accounts for only 1-3% of all tuberculosis cases worldwide and in the United States [2,3]. It usually affects major weight-bearing joints such as the spine, hip, and knee. Hand involvement is extraordinarily rare in adults, particularly in the fingers, constituting less than 1% of all skeletal tuberculosis cases [2,4].

In adults, TB osteomyelitis usually affects long bones like the femur, but when the hands are involved, it can be misdiagnosed as a post-traumatic infection or another inflammatory condition. The nonspecific symptoms and slow disease progression make a high index of suspicion essential for an accurate and timely diagnosis.

The onset of osteomyelitis in the small bones of the hand is not well-documented in the existing literature. TB osteomyelitis usually progresses slowly over several months. This report highlights a rare case of extrapulmonary tuberculosis in an immunocompetent adult, presenting as osteomyelitis of the fifth metacarpal. Both the unusual location and the rapid progression contributed to its resemblance to common pyogenic infections, which can delay diagnosis.

## Case presentation

A 22-year-old man from Mexico, residing in the United States for 3 years, with no significant past medical history, presented to the emergency department with pain and swelling in his right hand. He reported trauma a week earlier when a wooden stick fell on his hand. He denied fever, chills, night sweats, weight loss, cough, shortness of breath, gastrointestinal symptoms, or drainage from the hand. He was married, had no pets at home, and had no known exposures to tuberculosis.

**Figure 1:**
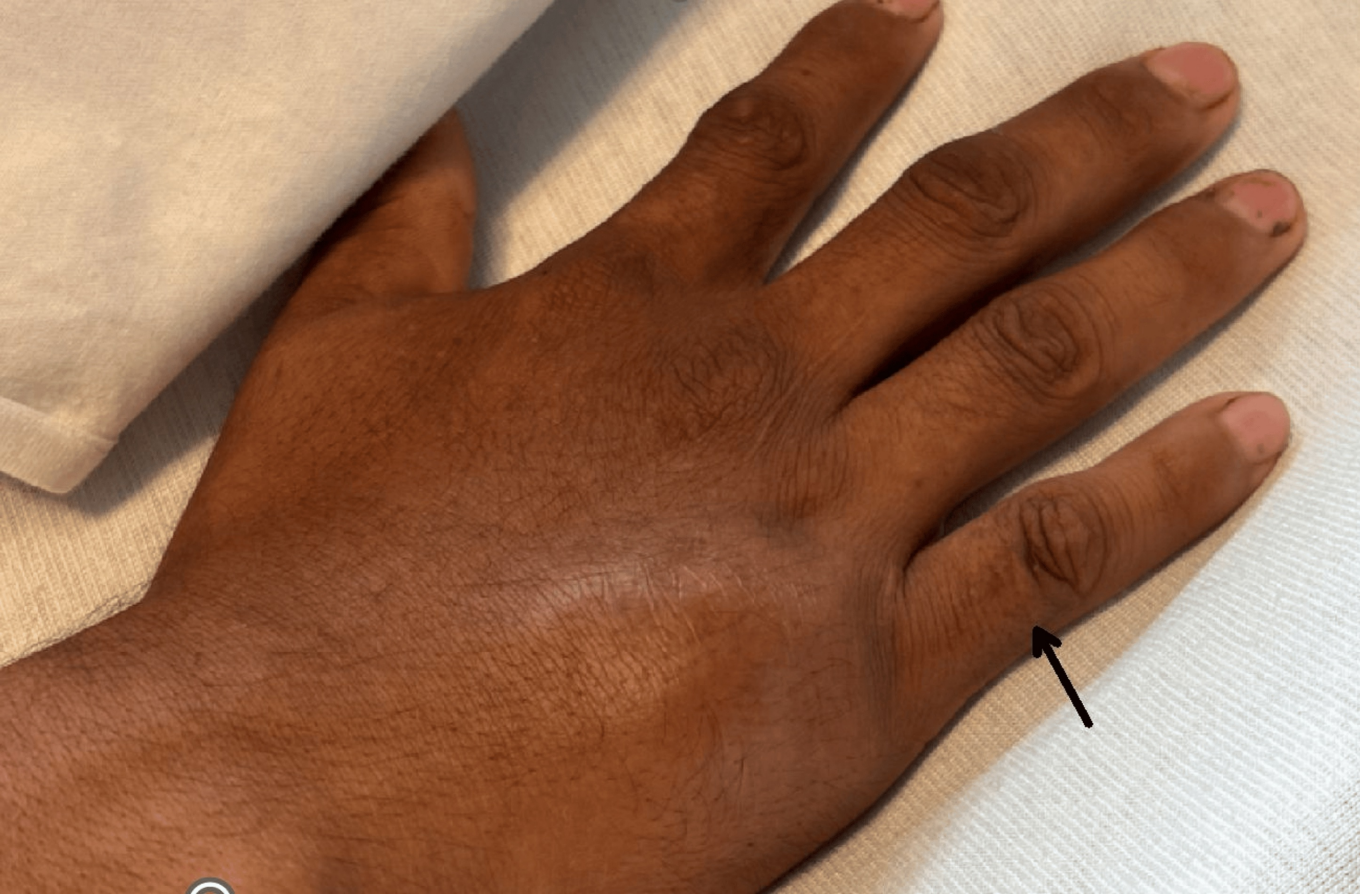
The fifth metacarpal of the right hand showed a small, diffuse, swollen area (arrow)

On examination, the lateral aspect of the fifth metacarpal of the right hand showed a small, diffuse, swollen area, tender to palpation, mildly erythematous, and warm, without fluctuance, streaking, cyanosis, clubbing, or edema (Figure 1).

There was a limited range of motion. No generalized lymphadenopathy was noted. Vital signs were normal with a temperature of 98.8° Fahrenheit, heart rate of 67, and blood pressure of 128/77.

His initial laboratory studies showed no anemia, no leukocytosis, and no platelet abnormalities. His peripheral smear appeared normal with no atypical cells. Renal, liver, and electrolyte assessments were unremarkable. CRP was 5.34 mg/L (normal range: 0-5 mg/L), and erythrocyte sedimentation rate was 88 mm/h (normal range: 0-20 mm/h). HIV, VDRL ( venereal disease research laboratory), hepatitis B, and hepatitis C tests were negative. Three separate sputum acid-fast bacilli (AFB) smears and PCR tests were negative, but the QuantiFERON TB Gold test was positive.

**Figure 2:**
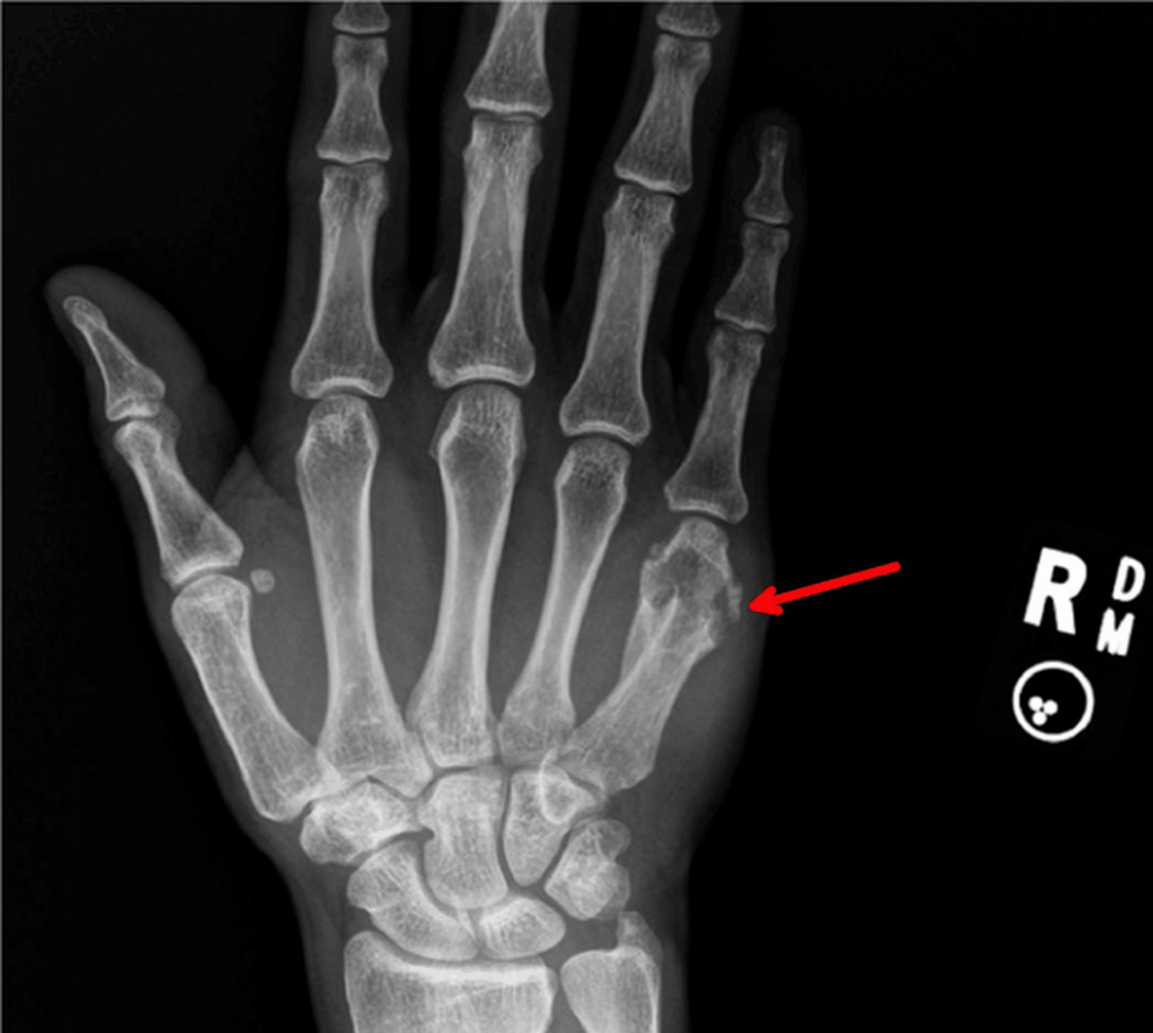
X-ray of the right hand shows lytic changes in the distal fifth metacarpal (arrow)

Imaging studies revealed significant pathology. An X-ray of the right hand showed a chronic deformity and lytic changes in the distal fifth metacarpal (Figure 2).

**Figure 3:**
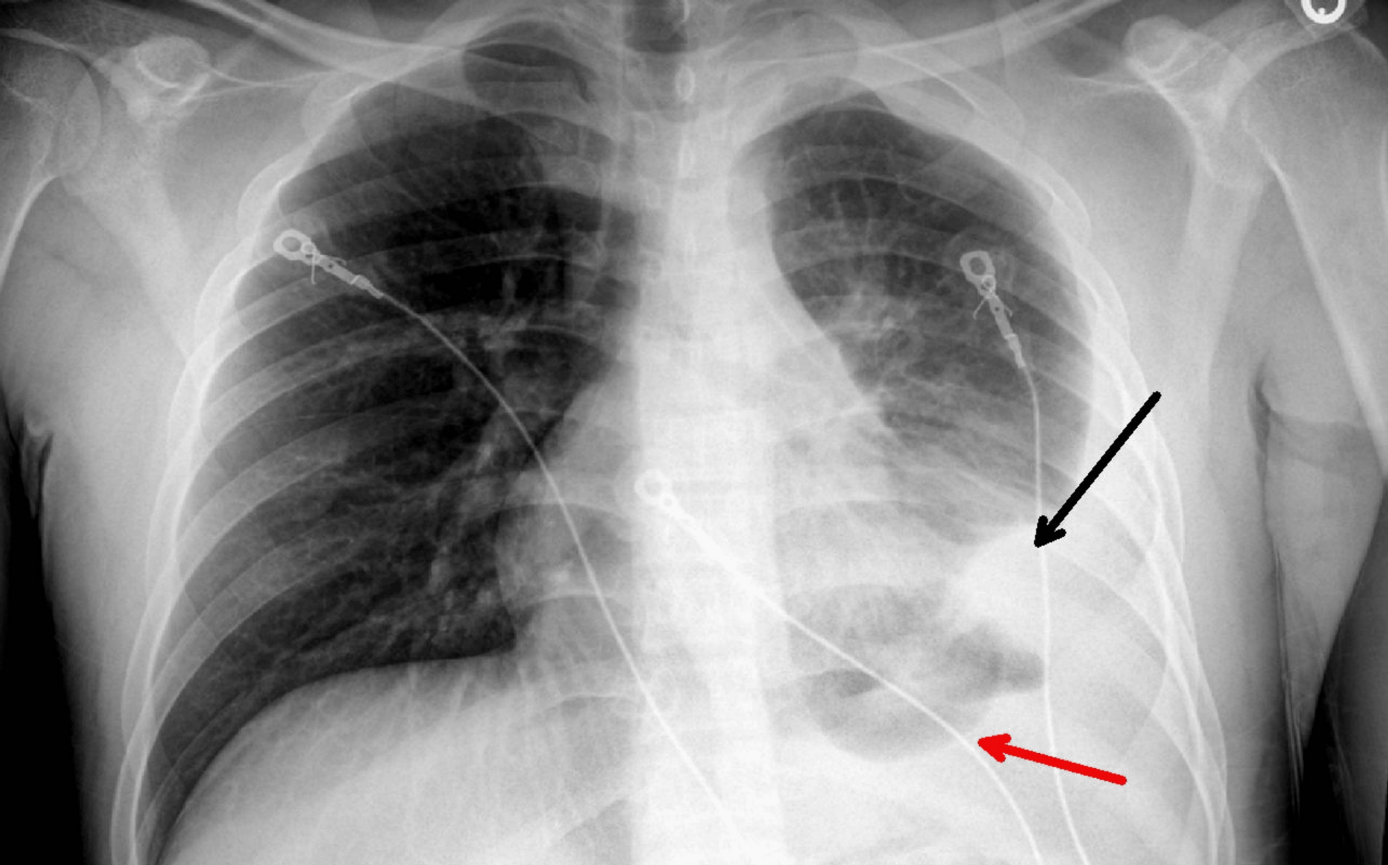
Chest X-Ray showing atelectasis (black arrow) and pleural effusion (red arrow) in the left lung.

A chest X-ray indicated left basilar airspace disease with an associated pleural effusion and atelectasis (Figure 3).

**Figure 4:**
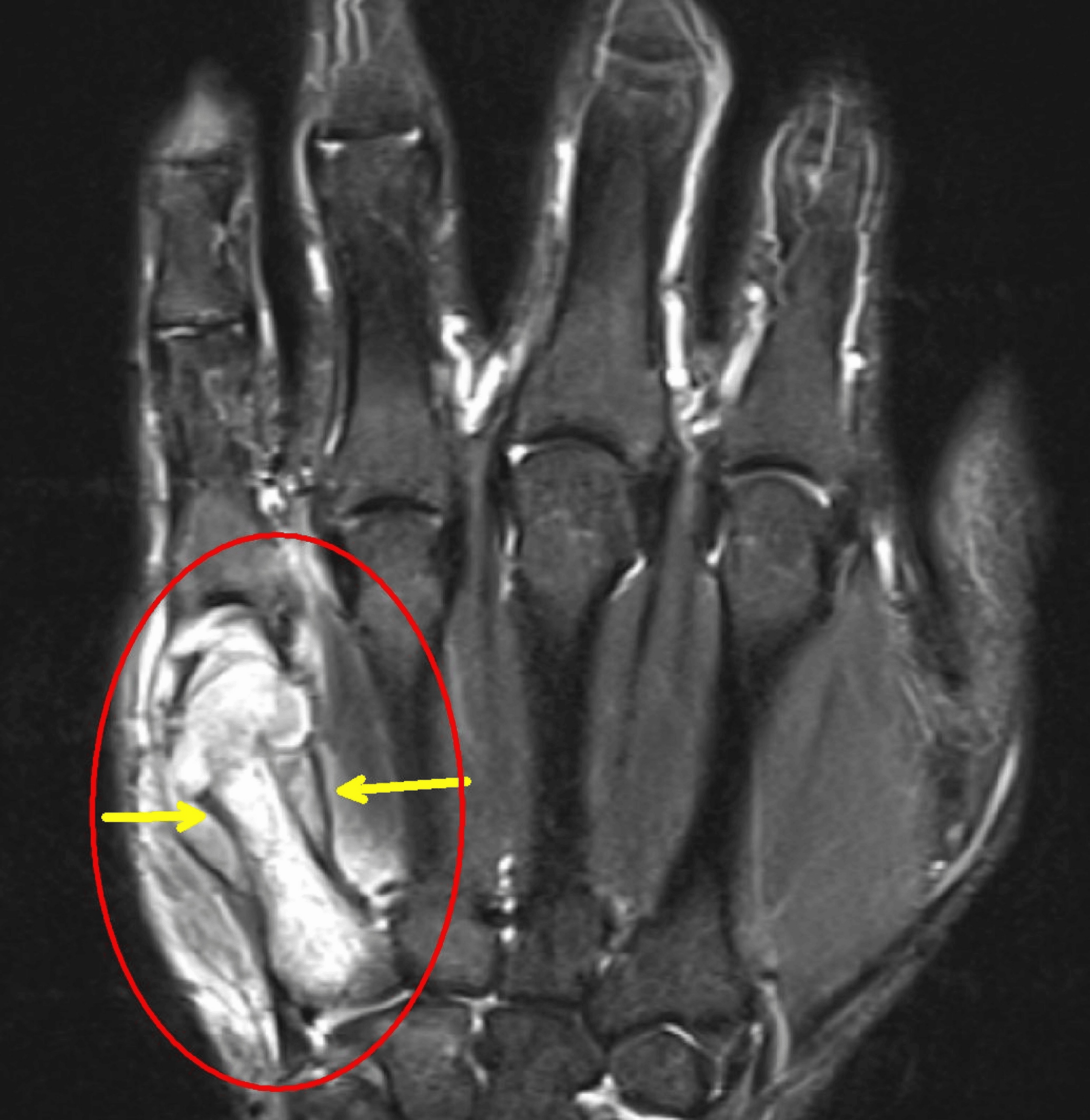
MRI of the right hand shows osteomyelitis in the fifth metacarpal bone (red circle) and an abscess lifting the periosteum (the two dark lines medial and lateral to the shaft, shown by the yellow arrows

CT scans of the chest, neck, abdomen, and pelvis identified a circumferential necrotic mass in the left pleura, causing compressive atelectasis, along with left supraclavicular and right axillary lymphadenopathy. They also demonstrated diffuse mural thickening of the small bowel, trace ascites, and peritoneal stranding. MRI of the right hand detected multiple contiguous abscesses around the fifth metacarpal. Additional findings included an intramedullary abscess, osteomyelitis throughout the metacarpal, and synovitis of the MCP joint (Figure 4).

**Figure 5:**
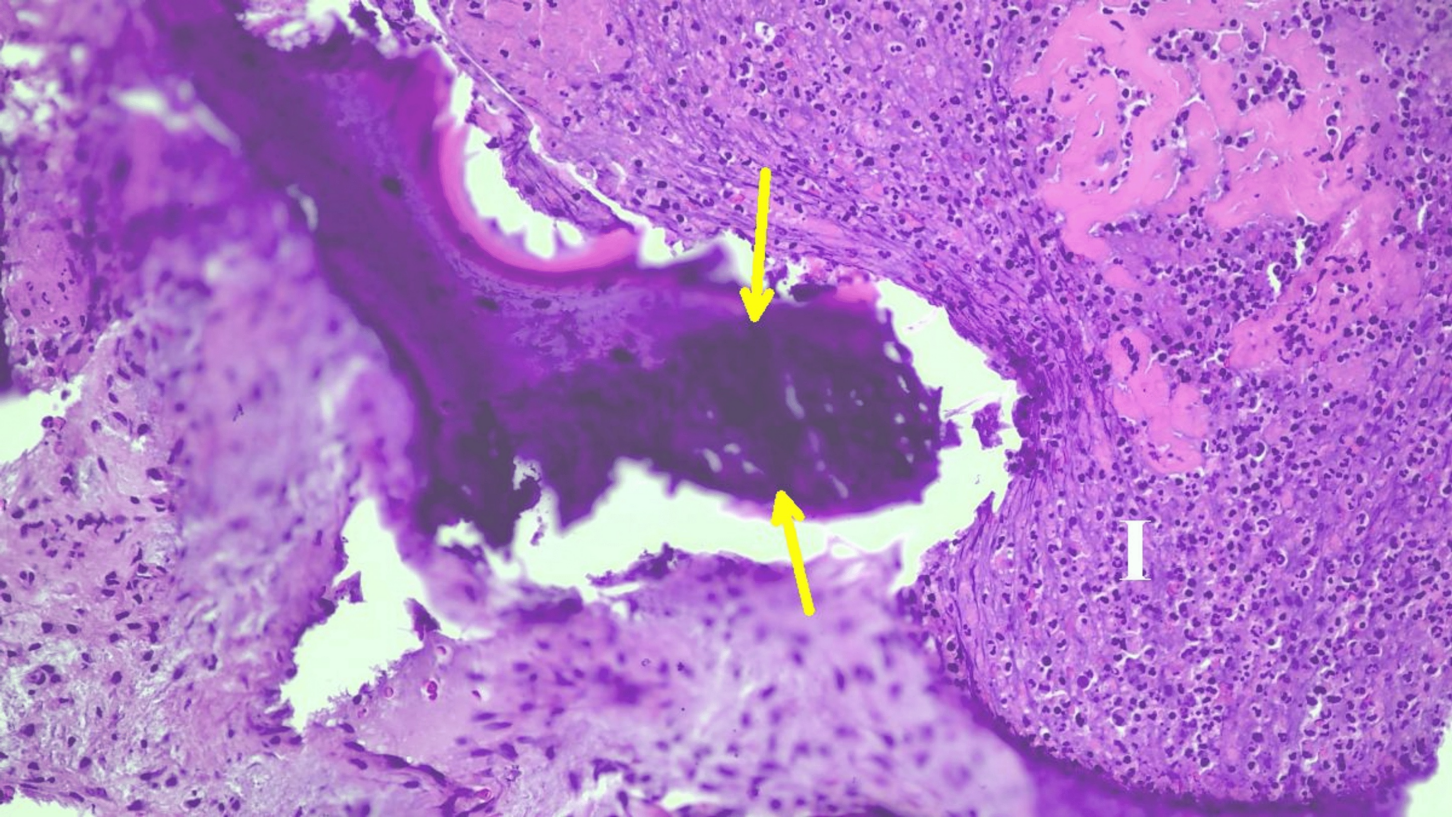
Hematoxylin and eosin (H&E)-stained section of the surgical debridement specimen of the right hand lesion (200x magnification), showing evidence of acute osteomyelitis Histopathologic features include bone trabeculae exhibiting irregular contours (yellow arrow) due to the surrounding neutrophilic granulocyte infiltrate with abscess formation (letter “I”).

The patient underwent surgical debridement of the lesion on the right hand. Bone and soft tissue samples were sent for aerobic, anaerobic, fungal, and mycobacterial stains and cultures, as well as for histopathology. The pathology showed acute osteomyelitis with abscess formation (Figure 5).

Testing revealed a positive AFB stain and a positive *Mycobacterium tuberculosis* PCR from the tissue. All bacterial, anaerobic, and fungal cultures remained negative.

A pleural biopsy was canceled because the patient refused. Thoracentesis was not performed due to the small volume of pleural fluid seen on ultrasound, as evaluated by the pulmonary team. Multidisciplinary discussions with Infectious Diseases, Orthopedic Surgery, and Pulmonary Medicine guided ongoing management. Airborne isolation continued until laryngeal tuberculosis was ruled out. 

The patient remained hemodynamically stable and afebrile throughout hospitalization. The clinical course, imaging results, and positive tissue PCR confirmed the diagnosis of tuberculous osteomyelitis of the fifth metacarpal with suspected extrapulmonary/pleural tuberculosis.

Discharge from the hospital was arranged for outpatient follow-up with the public health department. Antituberculous therapy was initiated with fixed-dose combinations of isoniazid, rifampin, ethambutol, and pyrazinamide.

## Discussion

Limited literature exists on the timing of osteomyelitis onset in small bones; however, osteoarticular tuberculosis typically progresses over months and can lead to joint destruction, which often becomes clinically apparent late. This case report describes a unique presentation of extrapulmonary tuberculosis in an immunocompetent adult, manifesting as osteomyelitis in the fifth metacarpal--an uncommon location and progression timeframe. This rarity, combined with a presentation that mimics common pyogenic infections, often results in diagnostic delays [5]. The patient’s initial history of minor trauma and the absence of constitutional symptoms were misleading. Imaging raised suspicion for post-traumatic osteomyelitis or malignancy, with radiographs showing lytic lesions and MRI revealing abscesses, sinus tracts, and extensive osteomyelitis. Histopathology showed bone with abscess and acute osteomyelitis, but no identifiable organisms. A definitive diagnosis of *Mycobacterium tuberculosis* requires molecular testing of surgical tissue, emphasizing the importance of comprehensive microbiologic studies such as AFB smear, culture, and nucleic acid amplification for suspected atypical infections.

Although extremely rare, TB in the digits has been reported. A recent case involved TB of the second metacarpal of the left index finger in an adult from a lower socioeconomic group in India [6]. Both that case and ours required PCR technology for tissue samples to establish a diagnosis. The index finger case, however, did not have evidence of pulmonary involvement, did not undergo MRI, but presented with a draining local pustular lesion. For both cases, the differential diagnosis was broad and included pyogenic osteomyelitis, especially due to *Staphylococcus aureus* following trauma, as well as fungal infections such as histoplasmosis and coccidioidomycosis. Nocardiosis was also considered, given the patient's history of wood-related trauma and the initial finding of AFB on staining, as Nocardia species are weakly acid-fast and often linked to environmental inoculation [7]. Malignancies, including lymphoma or metastatic disease, were also strongly considered because of the patient's pleural and lymph node involvement [8].

However, subsequent tissue testing confirmed TB as the unifying diagnosis.

From a public health perspective, this case highlights TB trends in the United States, where roughly two-thirds of cases occur among foreign-born individuals (4). Recognizing uncommon TB signs in this group is crucial, as delayed diagnosis can lead to increased morbidity. Managing skeletal TB requires prolonged multidrug therapy, typically lasting 9-12 months [9]. 

As demonstrated here, surgical intervention plays both diagnostic and therapeutic roles. Initiating antituberculous treatment early is essential for preventing disease progression and preserving function.

## Conclusions

TB should be included in the differential diagnosis of acute osteomyelitis, especially in people from endemic regions. Radiologic and histologic findings are often nonspecific, making microbiologic and molecular testing essential. Early diagnosis and treatment are vital to improve outcomes.
